# Management perspectives on research contributions to practice through collaboration in the U.S. Veterans Health Administration: QUERI Series

**DOI:** 10.1186/1748-5908-4-8

**Published:** 2009-02-26

**Authors:** Thomas J Craig, Robert Petzel

**Affiliations:** 1 Office of Quality and Performance (retired), Veterans Health Administration, U.S. Department of Veterans Affairs, Washington, DC, USA; 2 U.S. Department of Veterans Affairs Midwest Health Care Network, Minneapolis, Minnesota, USA

## Abstract

The Quality Enhancement Research Initiative (QUERI) is a unique quality improvement program designed to connect health services researchers to Veterans Health Administration (VHA) management and operations, as well as to provide the science and initiative for making change. Through this process, QUERI stakeholders have learned that success and impact in improving healthcare quality and outcomes largely depends on coordination and collaboration among numerous VHA programs and organizations working to develop and implement evidence-based clinical policies, practices, and quality improvement strategies. This Commentary discusses some of these collaborative efforts and perceived successes in achieving common goals from the viewpoints of two closely involved VHA Operations/Support stakeholders.

The Commentary is part of a *Series *of articles documenting implementation science frameworks and tools developed by the U.S. Department of Veterans Affairs Quality Enhancement Research Initiative (QUERI).

## Introduction

In 1998, the U.S. Department of Veterans Affairs (VA) created the Quality Enhancement Research Initiative (QUERI)–a bold step into uncharted territory called "implementation research," representing a new path toward healthcare quality improvement (QI) [1]. Conceived by Drs. Kenneth Kizer, John Feussner and John Demakis of the Veterans Health Administration (VHA), the concept was to intimately connect health services researchers to VHA management and operations, and provide the science and initiative for making change. QUERI was uniquely positioned within VA's healthcare system to form the collaborative relationships necessary at a national and regional level. Through QUERI, health services researchers would work to: identify evidence-based best practices for diseases and conditions that are prevalent among veterans; analyze actual practice to identify deviations in quality/performance from best-practices; and then develop, implement and evaluate improvement programs to eliminate those gaps. Additionally, the new model was to help shorten the time span from when something is known to be effective and when it is actually implemented through the entire healthcare system.

QUERI ultimately created a community of researchers committed both to improving quality and efficiency of VA care and to gaining insight on how to implement best practice throughout a large healthcare organization [1,2]. In addition, QUERI developed collaborative links with key elements of the organization equally invested in quality improvement efforts, such as VA's Office of Quality and Performance (OQP), Patient Care Services (PCS), and the Office of Information (OI). Given the needs of these stakeholders, QUERI investigators explored relevant benefits and challenges, especially in relation to cost-effectiveness of implementation of various interventions at the facility and regional level. Through these efforts, QUERI and key stakeholders learned valuable lessons that allowed the type of progress described in the QUERI Series, as well as the challenges that had to be addressed [1-4].

QUERI has learned, for example, that success and impact in improving healthcare quality and outcomes largely depend on coordination and collaboration among the numerous VHA programs, and organizations working to develop and implement evidence-based clinical policies, practices, and quality improvement strategies. This Commentary discusses some of these collaborative efforts and their contribution to achieving common goals. Having been closely affiliated with the QUERI program by serving on its key guiding/advisory committee [2] – while simultaneously serving in different capacities within VHA operational or support positions, we have had the opportunity to observe how this Initiative has worked from a unique perspective.

## Strengthening collaborations within the Veterans Health Administration

Since the establishment of QUERI, there has been a strong and mutually productive partnership between QUERI and the VHA Office of Quality and Performance (OQP). Perhaps the most fundamental linkage between QUERI and OQP, as well as other national-level offices in the VA, has been the extensive cross-membership of QUERI staff on OQP committees and vice versa [2]. In addition, the expertise and research findings of the QUERI groups have contributed greatly to the success of several core OQP missions, including the development, implementation and evaluation of evidence-based clinical practice guidelines (CPGs), the development of clinical performance measures (PMs), and the institution of a quality improvement program based, in part, on QUERI research.

Conversely, national-level operational activities have contributed to the breadth and scope of QUERI investigations, such as providing access to quality improvement data for use in generating research. In this way, the partnership between QUERI and national-level offices can be seen as one in which QUERI research identifies evidence-based practices that can be used for the development of clinical guidelines, eventually leading to the institution of evidence-based performance measures for operational QI programs undertaken by central and field VA operations. In turn, the results of these quality improvement programs may be evaluated by QUERI and other related QI researchers to identify evidence and performance gaps that can lead to further QUERI-initiated research. The results of this research inform the development of new clinical performance guidelines and performance measures, creating a highly sophisticated form of total quality improvement. This Commentary outlines concrete ways in which this and other partnerships and collaborations are perceived to have contributed to the progress and targeted improvements in quality of care within VHA, as documented in a variety of published reports [1,3,4].

## Clinical practice guideline development, implementation and evaluation

A key activity of OQP over the past decade has been the development and dissemination of evidence-based clinical practice guidelines (CPGs) that address the most highly prevalent and costly conditions affecting the veteran population, e.g., heart disease and diabetes. Since the initiation of QUERI, this process has been enriched by participation of designated QUERI Centers – the decentralized operational arms of the QUERI Program [2], whose focus on specific conditions dovetails with the major VA CPGs. For instance, there are QUERI Centers devoted to chronic heart failure, diabetes, and HIV/hepatitis, as well as ischemic heart disease, mental health, polytrauma and brain-related injury, spinal cord injury, stroke, and substance use disorders. As a result, QUERI leaders have taken a key role as experts for the respective CPGs and have helped broaden the scope of the national CPG effort to include implementation and evaluation of the use of clinical practice guidelines in VHA.

In the QUERI Series, for example, Goetz et al discuss the development of an implementation intervention that relied on clinical reminders to improve recommended screening rates for HIV among veterans [5]. The Centers for Disease Control and Prevention (CDC) data show that 25% of HIV-infected patients in the United States do not know their HIV-positive status. To confirm and extend these data, HIV-QUERI evaluated the rates of HIV testing in veterans seen in five VA facilities and found that between January 1999 and December 2004, only 30% of the 45,776 at-risk veterans had been tested for HIV infection. Following an HIV-QUERI implementation intervention that incorporated clinical reminders, audit/feedback, and organizational change, preliminary data showed a significant increase of at-risk veterans who were offered HIV testing at the VA sites where the project was implemented. This intervention relied heavily on the built-in quality improvement infrastructure in the VA, including the electronic medical record, clinical reminder software, and familiarity with performance measures.

## Performance measures

As noted in several of the papers in this Series, QUERI has affected the development of evidence-based performance measures (PMs) in several ways. First, through research, QUERI Centers have provided information that has resulted in changes in specific performance measures to reflect new knowledge. For example, Krein et al [6] describe the Diabetes Mellitus QUERI (DM-QUERI) Center's analysis of the timing of retinal eye examinations for veterans with diabetes. Their finding that an annual examination is not necessary for patients whose current eye exam is normal helped create a change of the PM that recommended exams every other year, which was eventually adopted by both VA and HEDIS (Healthcare Effectiveness Data and Information Set) – a tool used by more than 90% of America's health plans to measure performance in healthcare services. This new PM redirects the focus of scarce resources on those veterans with the highest risk and enhances cost-effectiveness.

Another example of QUERI's influence on performance measures includes the finding by Bradley et al [7] that the AUDIT-C (Alcohol Use Disorders Identification Test) screening instrument for alcohol misuse/disorders was preferable to older instruments (e.g., the CAGE). As part of this study, investigators with the Substance Use Disorders QUERI (SUD-QUERI) Center successfully implemented the new evidence-based screening program in more than 800 outpatient clinic sites nationwide, and 93% of VA outpatients were screened for alcohol misuse. These findings resulted in VA mandating the use of the AUDIT-C to meet the existing performance measure requiring alcohol screening for veterans.

Thus, QUERI steps that emphasize the identification of evidence-based practices and their implementation in routine clinical care have directly and indirectly affected VA's performance measures, as well as those of other healthcare organizations, resulting in improvements in the quality of care for veterans and the nation.

## Quality improvement initiatives

Perhaps the most direct impact that QUERI has had on the quality of care in VA's healthcare system has been through its contribution to national and local quality improvement efforts. Following are a few outstanding examples.

The Spinal Cord Injury QUERI (SCI-QUERI) Center used data collected by OQP's External Peer Review Program (EPRP) measuring rates of influenza and pneumococcal immunization among veterans with spinal cord injury and disease to identify gaps in care. For example, EPRP data indicated that national influenza vaccination rates for veterans with SCI between 1996 and 2001 had been improving but remained low. SCI-QUERI then developed a successful implementation program that was eventually rolled out to 23 VA SCI Centers and increased rates of both influenza and pneumococcal immunization [8]. Vaccination rates improved from about 26% in the late 1990s to 74% for influenza and 89% for pneumonia vaccines in 2007. This and other examples underscore the importance of the QUERI process, in which performance gaps in care are identified and strategies developed to help close these gaps.

Central to this process is the access QUERI centers have to data collection support from OQP. This access has been facilitated by the use of a Data Use Agreement process in which OQP data are available to or targeted for QUERI researchers. An example of the way this access has enhanced VA's ability to initiate quality improvement programs was illustrated by Bradley et al [7], which used OQP data on alcohol screening and follow-up to identify a gap in practice between screening and follow-up evaluation and care. Referenced above, these findings led to additions to the 2007 VA performance measures to increase effective follow-up and care in this area.

The Ischemic Heart Disease QUERI (IHD-QUERI) Center was a major leader in a quality improvement effort to enhance VA care for veterans with this disease. Cardiac catheterization and interventional procedures are primary therapy for IHD, and increased access to cardiac catheterization can lead to improved outcomes for veterans with IHD. There had been no mechanism to monitor and evaluate how such procedures are used in the VA healthcare system. Thus the Cardiovascular Assessment and Tracking System for Cardiac Catheterization Laboratories (CART-CL) was developed to address the critical need for a systematic, system-wide method for tracking the use of catheterization procedures. As of November 2007, 75 VA sites are participating in CART-CL – a collaborative effort between IHD-QUERI, VA's Patient Care Services, the Office of Quality and Performance, and the Office of Information.

The treatment of depression within VA's Primary Care is another example of QUERI's impact on the quality of veteran healthcare. This is an important area for QUERI focus because depression is the second most prevalent, chronic, disabling and costly illness in VA healthcare settings. Studies show that collaborative models for depression care delivery can cost-effectively bridge the gap between treatment effectiveness that is shown in research trials and the effectiveness actually achieved in primary care practice. Facilitated through the Mental Health QUERI (MH-QUERI) Center, VA's Translating Initiatives for Depression into Effective Solutions (TIDES) project [9] is an evidence-based collaborative approach to depression management. TIDES works to improve treatment adherence, promote symptom resolution, and prevent patient relapse by providing collaborative care. Such care begins when the primary care physician, using VA's computerized patient record system, refers veterans with symptoms of depression to a Depression Care Manager who fosters appropriate treatment. The implementation of TIDES at seven VA demonstration clinics enabled 8 out of 10 depressed patients to be treated effectively in primary care [9].

From one VA Network's experience [RP], the most striking feature of TIDES was the assistance provided by the research team in implementing system changes. QUERI researchers took staff step-by-step through the process of developing this program and provided education, assistance in hiring people, as well as measurement tools. This resulted in a very effective demonstration of how to appropriately implement and successfully make what was literally a profound change in the culture of how best to treat depression among veterans. A national roll-out effort is now being planned, and involves development of the close type of collaboration between QUERI researchers and operational stakeholders recognized in this commentary as essential to success [10].

In summary, from our viewpoint as VA managerial leaders closely aligned with QUERI, there are multiple examples of how QUERI has influenced the VA healthcare system, both directly and indirectly. In addition to those noted above:

• Chronic Heart Failure (CHF) QUERI reduced readmission rates for veterans with chronic heart failure by up to 10% in one VA network.

• IHD-QUERI improved lipid management for veterans with ischemic heart disease that translates into a commensurate reduction of about 75 coronary events over two years in one VA network.

• MH-QUERI increased the appropriate use of antipsychotics for veterans with schizophrenia that has led to a 10% decrease in costs of these drugs in one VA network.

• SUD-QUERI increased access to effective opioid agonist therapy for veterans with opioid dependence by 20%.

## Conclusion

The partnership between QUERI and other VA operational offices has resulted in successful development, implementation and evaluation of various evidence-based practices across the VA healthcare system. These efforts have helped to begin institutionalization of a cycle of quality improvement that can create a visible increase in the demonstrated quality of care through the effective implementation of evidence-based practice in routine care.

Because of the collaborative efforts between research and operations, we can cite several such quality improvement efforts in the diverse QUERI disease areas. To date, QUERI Centers have identified the research evidence and developed quality improvement interventions that have been implemented at VA's facility level, regional level, and even across regions. The next phase – system-wide national rollout – will be more challenging but will continue to require the collaborative efforts of many VA healthcare stakeholders.

QUERI has formed, and will continue to form the collaborative relationships necessary to address this challenge. The overarching goal remains the same: To systematically implement evidence-based practice across a large integrated healthcare system

## Authors' contributions

TJC and RP participated in all phases of development and revision of this manuscript. Both authors read and approved the final manuscript. The views expressed in the article are those of the authors, who are responsible for its contents, and do not necessarily represent the views of the U.S. Department of Veterans Affairs.
